# *Stat3*-mediated Th17 pathogenicity induced by periodontitis contributes to cognitive impairment by promoting microglial M1 polarization

**DOI:** 10.3389/fimmu.2025.1590665

**Published:** 2025-08-26

**Authors:** Yining Zhou, Xinyi Xie, Huiwen Chen, Lina Xu, Che Qiu, Hui Shen, Wei Zhou, Zhongchen Song

**Affiliations:** ^1^ Department of Periodontology, Shanghai Ninth People’s Hospital, Shanghai Jiao Tong University School of Medicine, Shanghai, China; ^2^ College of Stomatology, Shanghai Jiao Tong University, Shanghai, China; ^3^ National Center for Stomatology, Shanghai, China; ^4^ National Clinical Research Center for Oral Diseases, Shanghai, China; ^5^ Shanghai Key Laboratory of Stomatology, Shanghai Research Institute of Stomatology, Shanghai, China; ^6^ Department of Periodontology, Dental Disease Prevention and Treatment Center of Minhang District, Shanghai, China; ^7^ Laboratory of Oral Microbiota and Systemic Disease, Shanghai Ninth People’s Hospital, Shanghai Jiao Tong University School of Medicine, Shanghai, China

**Keywords:** periodontitis, cognitive impairment, neuroinflammation, Th17 cells, *Stat*3, microglia

## Abstract

**Introduction:**

Periodontitis has been identified as a potential risk factor for cognitive impairment associated with immune dysregulation. T helper 17 (Th17) cell-associated immune responses are involved in both diseases, while signal transducer and activator of transcription 3 (*Stat3*) is kown to be crucial for Th17 pathogenicity. Accordingly, in this study, we investigated how *Stat3*-mediated Th17 pathogenicity contributes to the link between periodontitis and cognitive impairment.

**Methods:**

Levels of Th17-related cytokines in gingival crevicular fluid (GCF) were measured in individuals with and without cognitive impairment. A periodontitis model was established in mice with conditional deletion of *Stat3* in Th17 cells (*Stat3* ^fl/fl^; *Il17a-*CreERT2, cKO) and wild type (*Stat3* ^fl/fl^, WT) mice via injection of *Porphyromonas gingivalis* lipopolysaccharide (*P. gingivalis* LPS) into gingival sulcus. Cognitive function was assessed through behavioral tests. Expression of Th17-related cytokines and microglial pro-inflammatory markers was evaluated by reverse transcription-quantitative PCR (RT-qPCR), ELISA, flow cytometry, and immunohistochemistry. To evaluate effects of CD4^+^ T cells on microglial M1 polarization, BV2 microglia were co-cultured with primary CD4^+^T cells which were stimulated with *P. gingivalis* LPS after isolated from cKO and WT mice.

**Results:**

Compared with cognitively normal participants, levels of Th17-related cytokines increased in participants with cognitive impairment. Significant alveolar bone resorption and cognitive impairment were observed in WT mice with periodontitis. These periodontitis-induced changes were alleviated in cKO mice, accompanied by a weakening of neuroinflammation and mitigation of Th17 immune responses. *In vitro*, M1 polarization and activation of the MAPK/ERK signaling pathway were inhibited in BV2 cells co-cultured with *Stat3*-deleted Th17 cells.

**Conclusion:**

*Stat3*-mediated Th17 pathogenicity bridged the correlation between periodontitis and neuroinflammation related to cognitive impairment, offering novel perspectives for a therapeutic target for blocking the mouth-to-brain axis.

## 1 Introduction

Periodontitis, triggered by plaque biofilm, is a chronic and progressive inflammatory disease affecting periodontal tissues (1). Evidence suggests that periodontitis not only results in the destruction of periodontal supporting tissues, but also exacerbates systemic diseases (2). Over past decade, a correlation between periodontitis and cognitive impairment has been reported. Meta-analyses have found that the incidence of cognitive impairment is significantly higher in patients with severe periodontitis than in periodontal healthy participants (3, 4). While epidemiological evidence strongly supports that periodontitis represents a significant risk for developing cognitive impairment, the underlying mechanism remains unclear.

Immune dyshomeostasis is regarded as a key factor linking periodontitis and cognitive impairment (5). It has been demonstrated that CD4^+^T cells can be neurotoxic and act as essential mediators of neuroinflammation (6, 7). CD4^+^T cells can differentiate into multiple subtypes, including T helper cells 1 (Th1), Th2, Th17 and regulatory T cells (Tregs) (8). Notably, Th17 cells have been proven to possess a greater ability to migrate toward the central nervous system (CNS) parenchyma than other T cell subpopulations, implicating that Th17 cells may be involved in promoting neuroinflammation (9, 10). Clinical studies have reported that the proportion of Th17 cells in serum is positively associated with cognitive impairment in patients with Alzheimer’s disease (11). Moreover, in mice, injection of *Porphyromonas gingivalis* lipopolysaccharide (*P. gingivalis* LPS) into the gingival sulcus induced learning and memory deficits, accompanied with an imbalance between Th17 and Treg cells (12). These results indicated that Th17 pathogenicity may be an important mediator in the pathogenesis of cognitive impairment and periodontitis.

Signal transducer and activator of transcription 3 (STAT3) is crucial for Th17 cell differentiation, and cell proliferation as well as for the expression of Th17-related cytokines (13, 14). Interleukin (IL)-23 and IL-6 can activate *Stat3*, which subsequently induces Th17 cell maturation and IL -17 secretion (15). Given that the overexpression of IL-17A stimulates the inflammatory cascade and disease pathogenesis, the inhibition of *Stat3* signaling is considered a potential therapeutic strategy for reversing the robust autoimmune responses via suppression of Th17 differentiation.

Considering that periodontal microbial dysbiosis was reported in patients with cognitive impairment (16), potentially leading to Th17 immune responses, we hypothesized that Th17 cells might connect periodontitis and neuroinflammation and that *Stat3* might be involved in the mouth-to-brain axis through modulating Th17 differentiation and function. Moreover, we sought to address the scarcity of data exploring the interactions between CD4^+^T cells and microglia in the CNS.

Therefore, levels of Th17-related cytokines in gingival crevicular fluid (GCF) from individuals with and without cognitive impairment were measured. Furthermore, we established an experimental periodontitis model in mice with the conditional knockout of *Stat3* in Th17 cells to evaluate the effects of Th17 pathogenicity on cognitive function. Additionally, we observed microglia pro-inflammatory responses toward CD4^+^T cells mediated by *Stat3* through a BV2-CD4^+^T cell coculture system. The aim of the current study was to explore the mouth-to-brain axis and provide novel insights into the role of Th17 pathogenicity in the connection between periodontitis and cognitive impairment.

## 2 Materials and methods

### 2.1 Participants and sample collection

Cognitively normal participants (CN group) were enrolled from the Department of Periodontology, Shanghai Ninth People’s Hospital from November 2019 to December 2021. Participants with cognitive impairment (CI group) were recruited from the Department of Neurology, Ruijin Hospital, and the Department of Geriatric Psychiatry, Shanghai Mental Health Center, from November 2019 to November 2021. Prior to participation, all the volunteers provided their written informed consent. The sample size was calculated using PASS 15. This study was approved by the Research Ethics Committee of Shanghai Ninth People’s Hospital, and related hospitals (Ethical consent No. SH9H-2019-T178-1).

Inclusion criteria: (i) Individuals diagnosed with Alzheimer’s disease dementia, supported by magnetic resonance imaging results. The diagnosis adhered to the guidelines set in 2011 g (17); (ii) Individuals who were cognitively normal, with a Montreal Cognitive Assessment (MoCA) score of ≥ 26 (18); (iii) Functional tooth number of ≥ 6; (iv) Participants who were able to cooperate during periodontal examination.

Exclusion criteria: (i) Functional tooth number of < 6; (ii) Received periodontal treatments within the last 6 months before sampling; (iii) Had open surgical treatments of the head and/or mouth; (iv) Individuals with acquired immune deficiency syndrome, hepatitis B infection, or other infectious diseases; (v) Individuals with diabetes mellitus; (vi) Those with a history of cancer, inherited metabolic diseases, hormone-dependent diseases, radiotherapy, and chemotherapy; (vii) Those administered antibiotics, immunomodulators, cytokines, and probiotics within the last 3 months.

Two periodontists were responsible for the full-mouth periodontal examination of participants. The number of teeth, periodontal probing depth (PPD), clinical attachment level (CAL), percentage of CAL > 3 mm (CAL > 3 mm%), and percentage of bleeding on probing (BOP%) were evaluated in the examination. Data consistency of periodontal examination was assessed by the Kappa coefficient and inter-class correlation coefficients.

GCF samples were collected from the Ramfjord index teeth and preserved following the procedures as previously described (16). A 150 µL volume of assay buffer was added to each sample, which contained ten paper strips. The supernatant was harvested and analyzed using multiplex cytokine assay (Luminex, UNIV, China) and ELISA analysis (EHC009, Neobioscience, China; EHC107b, Neobioscience, China; JL19287, Jianglai, China) which was conducted according to the manufacturer’s instructions.

### 2.2 Mice

Mice with a conditional deletion of *Stat3* in Th17 cells (*Stat3* ^fl/fl^; *Il17a-*CreERT2, cKO) were generated by breeding *Stat3* ^fl/fl^ mice (B6.129S1-*Stat3* ^tm1Xyfu^/J) with *Il17a-*CreERT2 mice (C57BL/6JSmoc-*Il17a* ^em1(2A-CreERT2)Smoc^), obtained from the Nanjing University Institute of Biomedical Sciences (Nanjing, China). Littermate *Stat3* ^fl/fl^ mice served as wild type (WT) controls. At 6 weeks of age, both cKO and WT mice were administered Tamoxifen (Sigma-Aldrich, USA) at a dose of 100 mg/kg via intraperitoneal injection for 5 consecutive days. Genotype identification criteria was shown in **Supplementary Figure S1**.

### 2.3 Animal model

Mice (*n*=72; both male and female mice were used to minimize gender-related bias), aged 8–10 weeks, were randomly assigned to 4 groups as follows: (1) saline-treated WT group (WT-NS), (2) saline-treated cKO group (cKO-NS), (3) *P. gingivalis* LPS-treated WT group (WT-LPS), and (4) *P. gingivalis* LPS-treated cKO group (cKO-LPS). Mice in the WT-LPS and cKO-LPS groups were injected with *P. gingivalis* LPS (InvivoGen, France) into the palatal gingival sulcus of maxillary first molars twice a week for 4 weeks at a dose of 0.5 mg/kg, whereas mice in the WT-NS and cKO-NS groups were injected with a same dose of saline (19). Two days after the final administration, behavioral tests were carried out.

### 2.4 Micro-computed tomography scan and methylene blue staining

Maxillae excised from the mice were scanned by micro-CT (SkyScan 1272, Switzerland) at a 25 μm resolution, 70 kV and 142 μA. Bundled vendor software was used for three-dimensional reconstruction and data processing. Bone mineral density (BMD; g/cc), the distance from the enamel-cemental junction to the alveolar bone crest (CEJ-ABC; μm), and the percent bone volume (BV/TV; %) were analyzed to assess bone quality and resorption. The maxillary first molar area was selected as the region of interest (ROI).

The maxillae samples were fixed in 4% paraformaldehyde (PFA) for 24 h, and subsequently stained with 1% methylene blue. The palatal structures of the area between the maxillary first molar and second molar were observed under a stereomicroscope.

### 2.5 Behavioral tests

Open field test (OFT): The equipment consisted of a rectangular arena surrounded by walls that stood 300 mm high. Each mouse was softly positioned at the center of the arena, and their movements were recorded for a duration of 5 minutes. The total distance and the average speed were measured to evaluate initial neuromotor function.

Morris water maze (MWM): The apparatus (Datum Mobile, China) of MWM was composed with a circular pool (120 cm in diameter and 50 cm in depth) and a platform (9 cm in diameter). The mice were slightly put into the pool with their heads facing toward the wall. They were given 90 s to learn how to utilized the visual cues surrounding the pool to locate the invisible platform. Each mouse underwent training 4 times every day for 5 consecutive days. On the sixth day, the platform was removed and a probe test was conducted, during which the mice swam twice in the two quadrants away from the platform (20). Latency (the time of each mouse to find the platform), number of platform crossings (the frequency of each mouse crossing the area where the platform had previously been located on day 6, and accumulative time spent in the target quadrant of each mouse were analyzed.

Passive avoidance test (PAT): was carried out in an instrument with a light zone and a dark zone, which were separated by a retractable door. On the first day of the test, the mice were positioned in the light area for 10 s until the door opened. Once completely entering the dark zone, the mice received a mild shock of 36 V. Same process was carried out on the next day. The latency to enter the dark zone and the error times (the number of electrical shocks) in 5 min were recorded.

EthoVision XT (Noldus Information Technology, the Netherlands) was used for the record and analyses for all behavioral tests.

### 2.6 Flow cytometry

Mouse brain tissues were mechanically ground and myelination was removed by Percoll (GE Healthcare Life, Sweden) to prepare single cell suspension adhering to a previously described protocol (21, 22). Lymphocytes were separated from spleen tissues using erythrocyte lysis buffer.

The cells were resuspended in RPMI-1640 medium containing 10% fetal bovine serum, 100 U/mL penicillin, PMA (20 ng/ml) (P1585, Sigma-Aldrich, USA), ionomycin (1 μM) (HY-13434, MedChemExpress, China) in the presence of Golgiplug (555029, BD Pharmingen, USA) and incubated at 37°C, 5% CO_2_. After 5 h restimulation, the cells were collected and anti-CD16/32 (553141, BD Pharmingen, USA) were used to prevent non-specific binding, then stained with a viability kit, as well as with antibodies against CD4 and CD25. Subsequently, cells were fixed and permeabilized using a Fixation/Permeabilization Kit (554714, BD Pharmingen, USA) following the manufacturer’s instructions. Finally, the cells were stained with intracellular cytokines IL-17A or Foxp3 to define Th17 cells or Treg cells. The data were analyzed by FlowJo software V10.

FACS antibodies: FVS-BV510 Zombie Aqua Fixable Viability Kit (423102, Biolegend, China); FITC-labeled anti-mouse CD4 (553651, BD Pharmingen, USA); Alexa Fluor^®^ 647-labeled anti-mouse IL-17A (560184, BD Pharmingen, USA); PE-labeled anti-mouse CD25 (553075, BD Pharmingen, USA); Alexa Fluor^®^ 647-labeled anti-mouse Foxp3 (560401, BD Pharmingen, USA).

### 2.7 Isolation and stimulation of CD4^+^ T cells

Primary CD4^+^ T cells of cKO and WT mice were selected from spleen via CD4 MicroBeads (130-117-043, Miltenyi Biotec, Germany), and cell purity (>95%) were confirmed by Flow cytometry (23). The isolated cells were resuspended in RPMI-1640 medium and plated into 12-well plates at a density of 10^6^ cells/mL in the presence of anti-CD3 (5 μg/mL) (16-0032-85, Invitrogen, USA), anti-CD28 (1 μg/mL) (16-0281-85, Invitrogen, USA) and IL-2 (10 ng/mL) (HY-P70646AF, MedChemExpress, China).

After 72 h of *in vitro* culture, the isolated CD4^+^ T cells were assigned into four goups: (1) WT group, (2) cKO group, (3) WT+LPS group, and (4) cKO+LPS group. WT+LPS group and cKO+LPS group were treated with *P. gingivalis* LPS (1 μg/mL) for another 24 h. Supernatants of the cell cultures were obtained as the conditional medium (CM) for BV2 cells and total RNA was extracted for subsequent experiments.

### 2.8 Co-culture of microglia

BV2 cells were cultured in DMEM (Gibco, USA) supplemented with 10% FBS, 100 U/mL penicillin and 100 mg/mL streptomycin, at 37°C in a 5% CO_2_ environment. Cells were assigned into five groups: (1) +WT CD4^+^T group, (2) +cKO CD4^+^T group, (3) +WT CD4^+^T+LPS group, (4) +cKO CD4^+^T+LPS group, and (5) +WT CD4^+^T+LPS+ERKi group. CM was mixed with microglial medium at a 1:1 ratio. BV2 cells in group (1) and (2) were treated with. BV2 cells in group (3), (4), and (5) were treated with CM obtained from CD4^+^ T cells with *P. gingivalis* LPS. BV2 cells in group (5) were added with an ERK inhibitor (20nM) (SCH772984, Selleck, USA).

### 2.9 Reverse transcription-quantitative polymerase chain reaction (RT-qPCR)

Total RNA was extracted from gingiva tissues, cortex tissues and cultured cells using TRIzol reagent (Takara, Japan) and was synthesized into cDNA(Takara, Japan). The quantification was performed by a real-time PCR detection system (Roche, Switzerland) in combination with 2×SYBR Green qPCR Master Mix. Relative expression levels were calculated by 2^-△△Ct^ formula. The primers used in the experiments are listed in **Supplementary Table S1**.

### 2.10 Enzyme-Linked Immunosorbent Assay (ELISA)

Plasma was separated from blood samples by centrifugation. Cortex protein was extracted by radioimmunoprecipitation assay (RIPA) lysis buffer (Beyotime, China). Protein quantification was determined and equal amounts of each sample were applied to ELISA analysis to measure levels of IL-17, IL-6, IL-1β, TNF-α and IL-10.

ELISA kits: Mouse IL-1β ELISA Kit (EMC001b, Neobioscience, China); Mouse TNF-α ELISA Kit (EMC102a, Neobioscience, China); Mouse IL-6 ELISA Kit (EMC004QT, Neobioscience, China); Mouse IL-17A ELISA Kit (JLW20251, Jonlnbio, China); Mouse IL-10 ELISA Kit (JL20242, Jonlnbio, China).

### 2.11 Immunohistochemical staining

Brain samples were obtained and fixed in 4% PFA, then dehydrated and embedded. 5 μm-thick hippocampal sections were prepared and incubated with anti-Ionized calcium-binding adapter molecule 1 (Iba1) (ARG63338, Arigo, Taiwan). HRP-labeled Donkey Anti-Goat (A0181, Beyotime, China) was used as the secondary antibody, and positive areas were marked by a DAB staining solution (Beyotime, China). Regions from the hippocampal dentate gyrus and region and cortex were visualized by a light microscope (Leica TCS SP2, Germany). To quantify microglial cells, at least 3 regions of equal size (10000 μm^2^) from 3 mice per group were quantified manually for Iba1-positive cells.

### 2.12 Immunofluorescence staining

Co-cultured BV2 cells, after fixation and blocking, were incubated with anti-CD86 (1:200) (13395-1-AP, Proteintech, China) overnight at 4 °C. The secondary fluorescent antibody (1:1000) (A-11005, Invitrogen, USA) were applied for 1 h on the next day, and DAPI was used for nuclear staining. The samples were visualized under a fluorescence microscopy (Leica TCS SP2, Germany). The mean fluorescence intensity (MFI) was determined with ImageJ software.

### 2.13 Western blot

Total protein was extracted from BV2 samples by RIPA lysis buffer and quantified. Equivalent quantities of protein were loaded and segregated in SDS-PAGE gels, then transferred to PVDF membranes (Millipore, USA). Following blocking for 1 h, the membranes were stained with primary antibodies overnight at 4 °C. On day 2, the secondary antibodies were applied and the results were detected (34577, ThermoFisher, USA).

Western Blot antibodies: anti-MAPK (4312, Cell Signaling Technology, USA); Anti-pMAPK (9211S, Cell Signaling Technology, USA); Anti-ERK (4695, Cell Signaling Technology, USA); Anti-pERK (4370, Cell Signaling Technology, USA); Anti-GAPDH (AF0006, Beyotime, China); HRP-labeled Goat Anti-Rabbit (AF0208, Beyotime, China); HRP-labeled Goat Anti-Mouse (AF0216, Beyotime, China).

### 2.14 Statistical analysis

The normality of the data was assessed with Shapiro-Wilk test. Two-way ANOVA and one-way ANOVA were carried out for comparisons among multi-groups. In cases where the data found to be non-normally distributed, Kruskal-Wallis test was performed. Comparisons between two groups were performed using independent *t* test or Mann-Whitney U test. A *P* value of <*0.05* was considered statistically significant.

## 3 Results

### 3.1 Clinical characteristics and levels of Th17/Treg-related cytokines in GCF of the participants

In total, 17 individuals were enrolled in the CN group and CI group respectively. Mean MoCA score in the CI group was 13.00 ± 5.03, which was lower than that in the CN group. Moreover, the severity of periodontitis in the CI group was markedly higher than that in the CN group (**Supplementary Table S2**). Logistic regression analysis using a dataset from an article previously published by our team identified increased CAL as a risk factor for cognitive impairment (OR=2.783, 95% CI: 1.064-7.278) (16).

**Figure 1:**
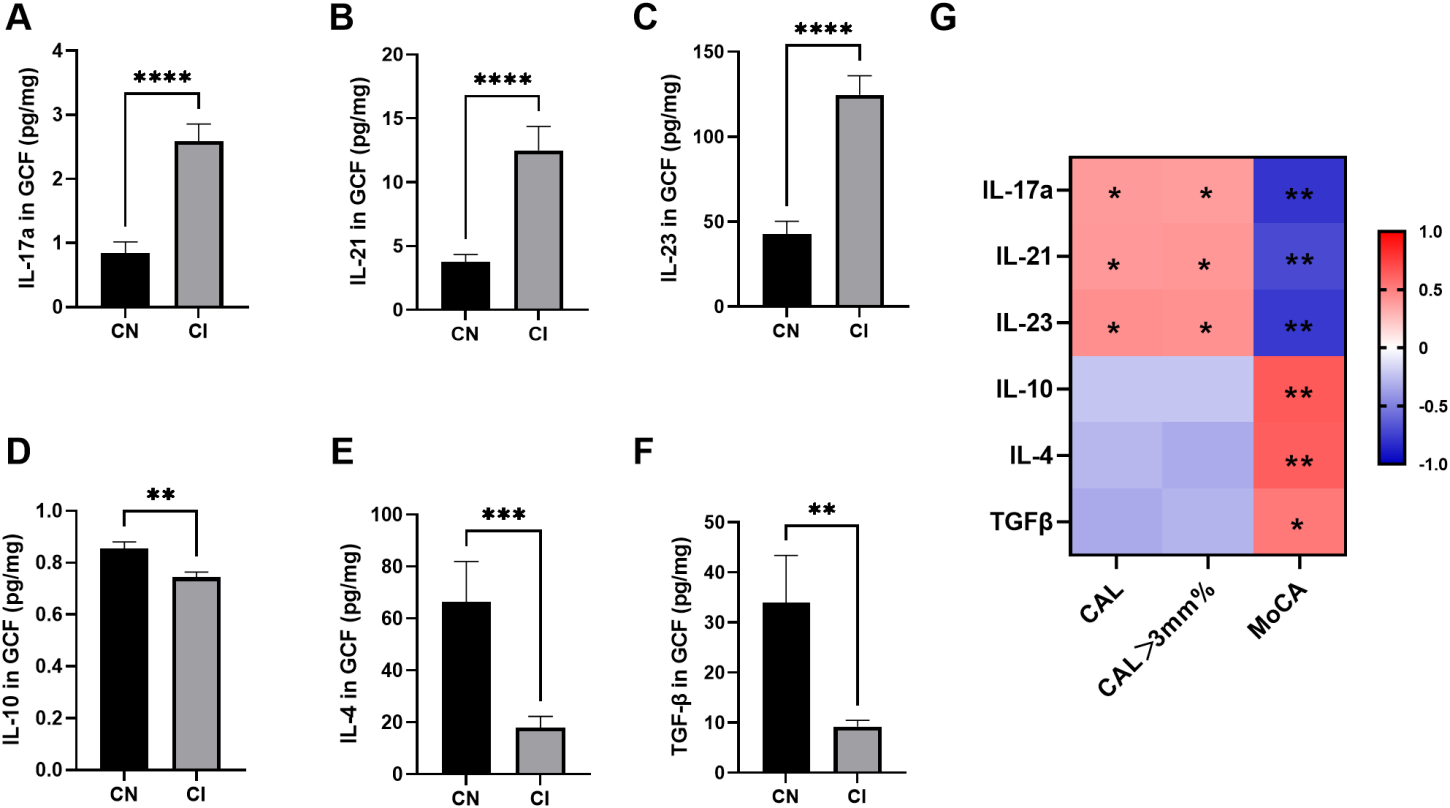
Levels of Th17/Treg-related cytokines in GCF from CN and CI groups (*n*=17 per group). **(A-C)** Th17-related cytokines (*IL-17A, IL-21* and *IL-23*) in GCF. **(D-F)** Treg-related cytokines (*IL-10, IL-4* and *TGF-β*) in GCF. Comparisons between groups were performed by Mann-Whitney U test in panels **(A-C, E, F)** and independent t test in panel **(D)**. **(G)** Spearman correlation analysis between cytokine levels and clinical indices. Blue and red classes denote negative correlation and positive correlation, respectively. *P < 0.05, **P < 0.01, ***P < 0.001, **** *P* < 0.0001. CN, cognitive normal participants; CI, participants with cognitive impairment; CAL, clinical attachment level; CAL > 3 mm%, percentage of CAL > 3 mm; MoCA, Montreal Cognitive Assessment.

The expression of Th17-related cytokines in GCF was higher in the CI group than in the CN group (**Figures 1A-C**), whereas that of anti-inflammatory cytokines displayed the opposite trend (**Figures 1D-F**). The levels of Th17-related cytokines, IL-17A, IL-21, and IL-23 were found to be positively correlated with CAL and CAL>3 mm% (**Figure 1G**). Conversely, MoCA scores exhibited a negative correlation with levels of Th17-related cytokines.

Therefore, it was suspected that periodontitis was associated with cognitive impairment, potentially involving immune responses mediated by Th17 cells.

### 3.2 Effects of *Stat3*-mediated Th17 cells on *P. gingivalis* LPS-induced periodontitis

**Figure 2:**
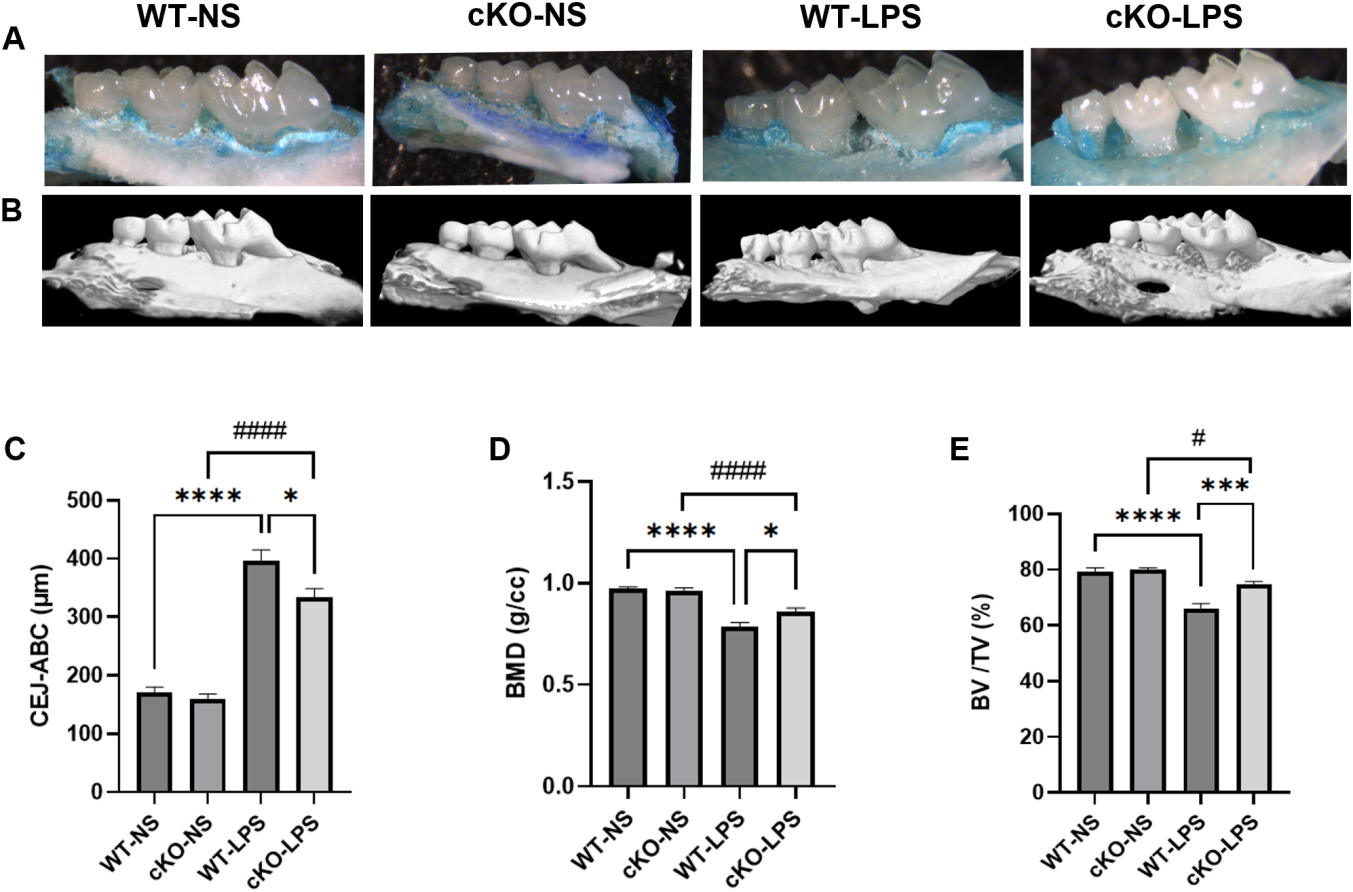
Effects of *P. gingivalis* LPS-induced periodontitis on alveolar bone resorption Methylene blue staining and micro-CT were used to show the palatal alveolar bone of the maxillae (*n*=10 per group). **(A)** Representative images of the maxillae from the palatal view staining by methylene blue. **(B)** Representative reconstruction images of three-dimensional palatal maxillae via micro-CT. **(C)** Quantification of CEJ-ABC. **(D)** Quantification of BMD. **(E)** Quantification of BV/TV. Data are presented as the means ± standard error of the mean. *P* values were determined by one-way ANOVA. *P < 0.05, ***P < 0.001, ****P < 0.0001 compared to the WT-LPS group; #*P* < 0.05, ####*P* < 0.0001 compared to the cKO-NS group. micro-CT, micro-computed tomography. CEJ-ABC, distance from enamel cementum junction to alveolar bone crest; BMD, bone mineral density; BV/TV, bone volume fraction.

To further investigate the role of Th17 cells in the association between periodontitis and cognitive impairment *in vivo*, an experimental periodontitis model was established in mice carrying Th17 cell-specific deletion of *Stat3*. Micro-CT revealed resorption of the alveolar bone in both WT-LPS group and cKO-LPS group. Representative images of methylene blue staining and three-dimensional reconstruction of the palatal alveolar bone of the maxillae were shown in **Figures 2A, B**. Compared to the WT-NS and cKO-NS groups, BMD and BV/TV decreased in both the WT-LPS and cKO-LPS groups, whereas the CEJ-ABC increased (**Figures 2C-E**). Moreover, periodontal destruction was alleviated in cKO-LPS group, as evidenced by decrease in CEJ-ABC and increase in BMD and BV/TV compared to WT-LPS group (**Figures 2C-E**).

Additionally, in gingival samples, the mRNA expression of pro-inflammatory cytokines (*Il1β, Il17a, Il6* and *Tnfα*) was lower in the cKO-LPS group than in the WT-LPS group, whereas that of IL-10 was higher (**Supplementary Figure S2A**), indicating that both periodontal destruction and gingivitis were mitigated in the absence of *Stat3* in Th17 cells.

### 3.3 Effects of *Stat3*-mediated Th17 cells on cognitive function in periodontitis mice

**Figure 3:**
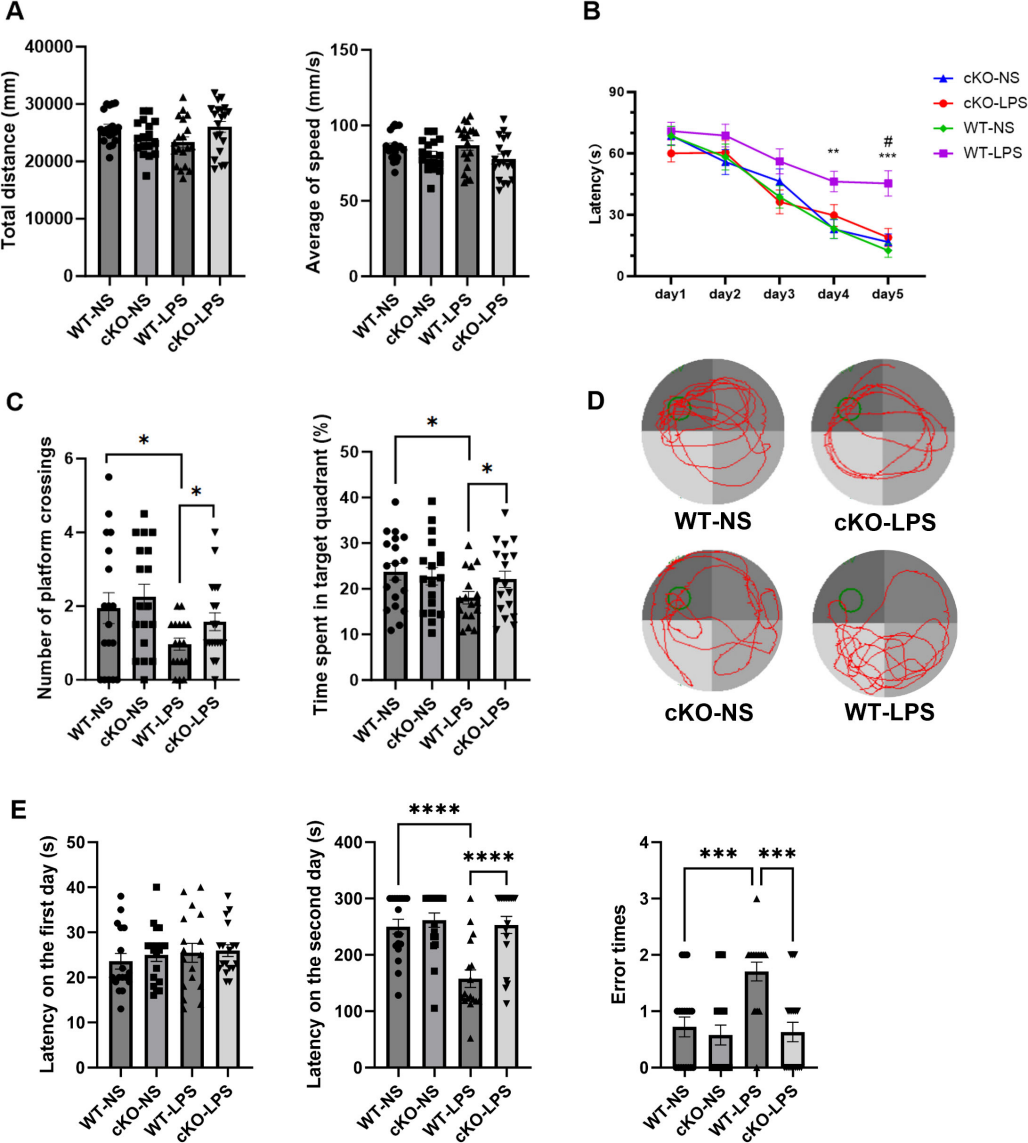
Effects of *Stat3*-mediated Th17 cells on cognitive function in periodontitis mice. The OFT was used to observe the locomotor activity of mice (*n*=17–19 per group). **(A)** Total distance covered and the average of speed in the OFT. MWM test was conducted to assess the spacial memory and learning ability (*n* = 17–19 per group). **(B)** Latency to find the platform during the training period. P values were determined by two-way ANOVA. **P < 0.01, ***P < 0.001 compared to the WT-NS group and #*P* < 0.05 compared to the KO-LPS group. **(C)** The number of platform crossings and the time spent in the target quadrant during the probe test on day 6. **(D)** Typical swimming trajectory during the probe test on day 6. *P* values were determined by one-way ANOVA. Data are presented as the means ± standard error of mean. *P < 0.05 compared to the WT-LPS group. The PAT was used to evaluate the learning ability (*n* = 17–19 per group). **(E)** The latency to enter the dark zone on day 1, the latency to enter the dark zone on day 2, and the number of errors. Data are presented as the means ± standard error of mean. *P* values were determined by one-way ANOVA. ***P < 0.001, ****P < 0.0001 compared to the WT-LPS group. OFT, open field test; MWM, Morris water maze; PAT, passive avoidance test.

The aforementioned results confirmed that the experimental periodontitis model was successfully established. Subsequently, behavioral tests were conducted to evaluate the influence of periodontitis on the learning and memory abilities. In the OFT, no behavioral differences were found among the four groups (**Figure 3A**), indicating that neither LPS treatment nor gene knockout disrupted the spontaneous activity of the animals.

In the MWM experiment, on day 4, the latency to find the platform was longer in the WT-LPS group than in the WT-NS group, whereas the cKO-LPS group exhibited a notably shorter latency than the WT-LPS group (**Figure 3B**). During the probe test on day 6, mice in the WT-LPS group showed fewer platform crossings and spent less time in the target quadrant than those in the WT-NS group. Notably, these changes were alleviated in the cKO-LPS group (**Figures 3C, D**).

In the PAT, the latency to enter the dark zone did not differ among the four groups on day 1. On the next day, mice in the WT-LPS group spent less time in the illuminated zone and made more errors in entering the dark zone compared with mice in the WT-NS and cKO-LPS groups (**Figure 3E**).

These findings suggested that periodontitis could impair the learning and memory abilities of mice, while deletion of *Stat3* in Th17 cells alleviated the negative effects of periodontitis on cognitive function.

### 3.4 Effects of *Stat3*-mediated Th17 cells on microglial M1 polarization in periodontitis mice

**Figure 4:**
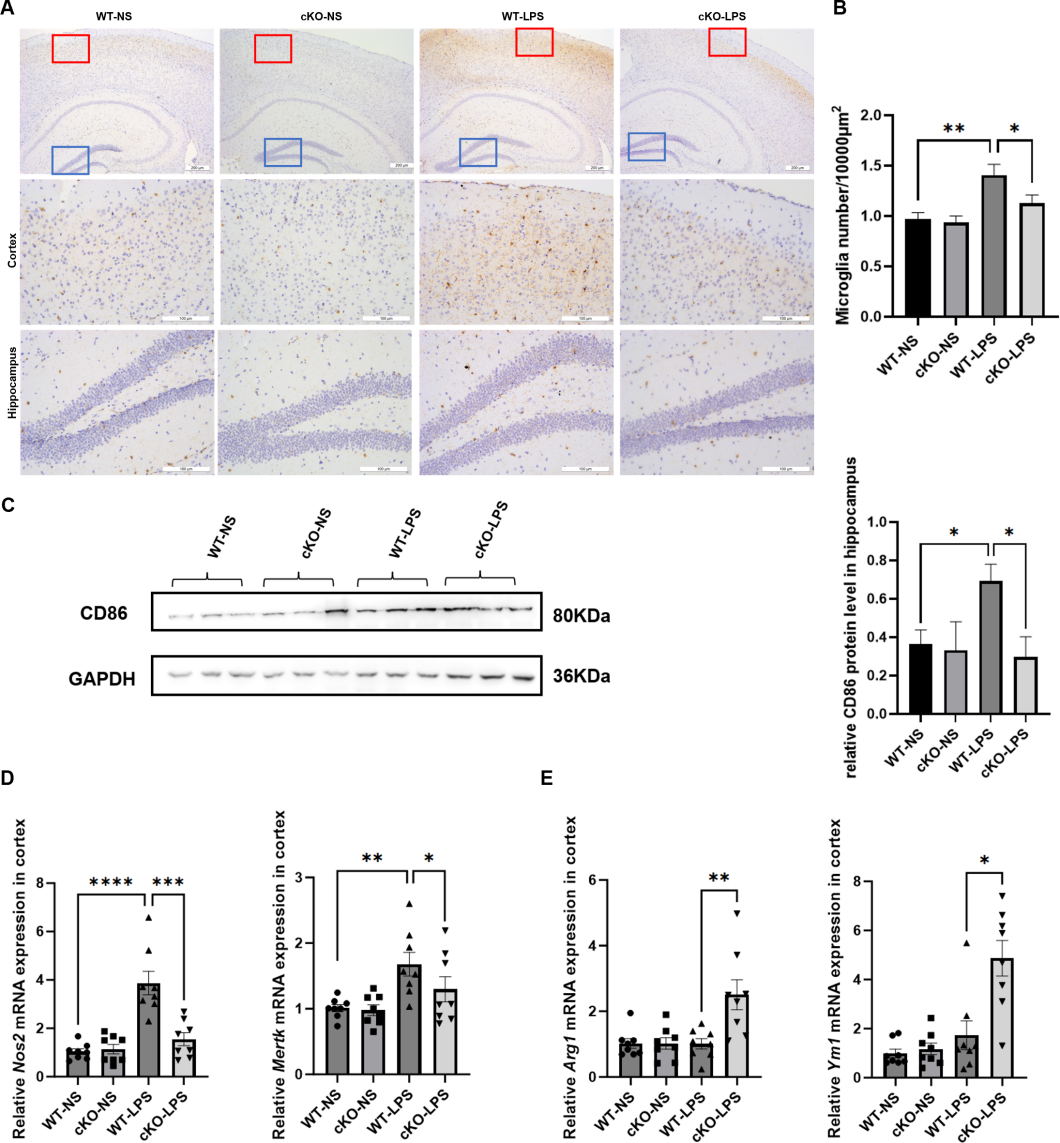
Effects of *Stat3*-mediated Th17 cells on microglial M1 polarization in periodontitis mice. **(A)** Representative images of Iba1-positive cells in cortex and hippocampus (Scale bar=200 or 100 µm). **(B)** Quantitative analysis of the density of microglia (*n*=3 per group). **(C)** Representative Western blot bands of CD86 in hippocampus and the quantitative analysis of CD86 protein in hippocampus. **(D)** mRNA expression of microglial M1 polarization markers (*Nos2* and *Mertk*) in cortex (*n* = 8 per group). **(E)** mRNA expression of microglial M2 polarization markers (*Arg1* and *Ym1*) in cortex (*n* = 8 per group). Data are presented as the means ± standard error of mean. *P* values were determined by one-way ANOVA. *P < 0.05, **P < 0.01, ***P < 0.001, ****P < 0.0001 compared to the WT-LPS group.

To further determine whether the experimental periodontitis-induced cognitive impairment observed in the study was related to neuroinflammation mediated by Th17 cells, the inflammatory responses of microglia was examined. As shown in **Figures 4A, B**, mice in the WT-LPS group showed an elevated microglial count. Moreover, the expression of microglial M1 polarization markers, CD86, Nos2, and MerTK, significantly increased in the WT-LPS group (**Figures 4C, D**), while that of M2 polarization markers, Arg-1 and Ym-1, decreased (**Figure 4E**). Importantly, these changes were partially reversed in the cKO-LPS group.

### 3.5 Effects of *Stat3* on Th17/Treg balance in periodontitis mice

**Figure 5:**
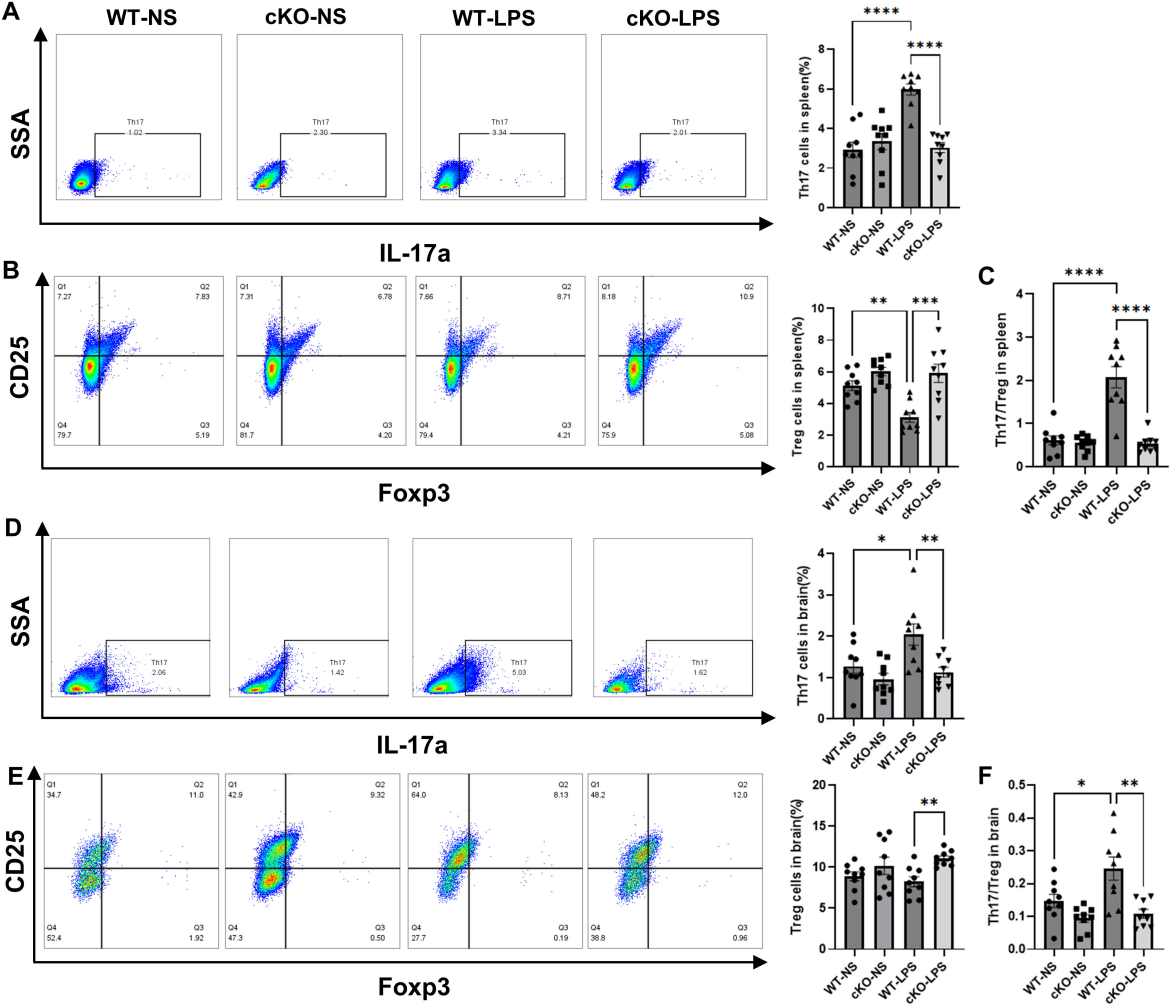
Effects of *Stat3* on Th17/Treg balance in periodontitis mice. The proportions of Th17 and Treg cells in CD4^+^T cells were assayed by flow cytometry (*n*=9 per group). **(A)** The proportion of Th17 in CD4^+^T cells in spleen. **(B)** The proportion of Treg cells in CD4^+^T cells in spleen. **(C)** The ratio of Th17/Treg in spleen. **(D)** The proportion of Th17 in CD4^+^T cells in brain. **(E)** The proportion of Treg cells in CD4^+^T cells in brain. **(F)** The ratio of Th17/Treg in brain. Data are presented as the means ± standard error of mean. *P* values were determined by one-way ANOVA. *P < 0.05, **P < 0.01, ***P < 0.001, ****P < 0.0001 compared to the WT-LPS group.

We further examined the effects of *Stat3* on T-cell differentiation and its correlation with systemic inflammation and neuroinflammation. The proportion of Th17 cells in CD4^+^T cells and the ratio of Th17/Treg in the spleen were significantly higher in the WT-LPS group than in the WT-NS group, whereas this Th17/Treg imbalance was mitigated in the cKO-LPS group (**Figures 5A-C**). Similar trends were found in brain tissues (**Figures 5D-F**).

Moreover, in plasma and cortex samples, the levels of pro-inflammatory cytokines increased in the WT-LPS group compared with those in the WT-NS group, while those of the anti-inflammatory cytokine, IL-10, decreased (**Supplementary Figure S2B, C**). These changes were markedly less pronounced in the cKO-LPS group, indicating that inflammatory responses in the peripheral and the CNS might be related to Th17/Treg imbalance induced by periodontitis.

### 3.6 Effects of *Stat3* in CD4^+^T cells stimulated by *P. gingivalis* LPS

**Figure 6:**
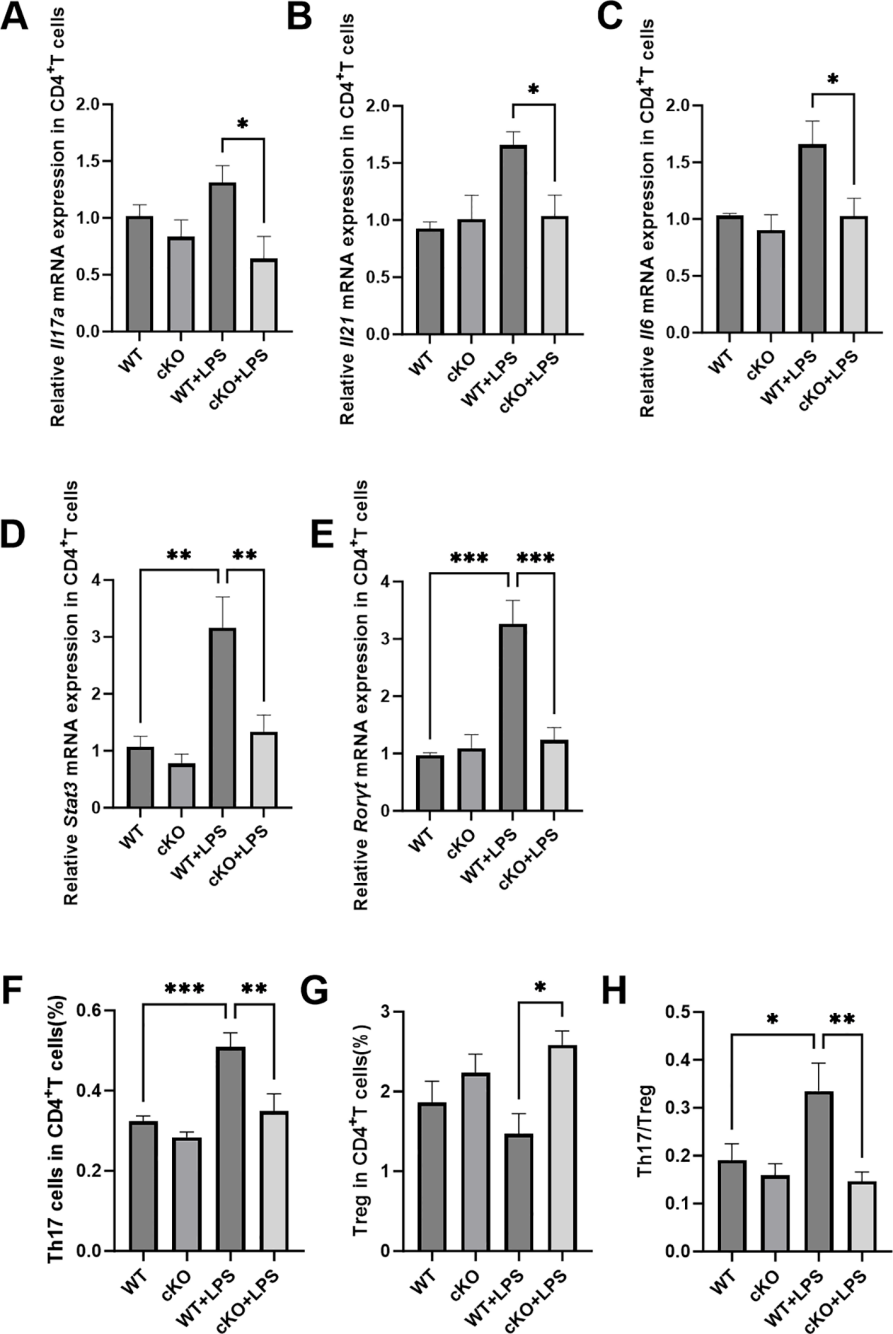
Effects of *Stat3* in CD4^+^T cells stimulated by *P. gingivalis* LPS. **(A-C)** mRNA expression of Th17-related cytokines (*Il17a, Il21* and *Il6*) in CD4^+^T cells (*n*=4 per group). **(D, E)** mRNA expression of Th17-related transcriptional factors (*Stat3* and *Rorγt*) in CD4^+^T cells (*n*=4 per group). **(F-H)** The proportion of Th17 and Treg cells in CD4^+^T cells (*n*=8 per group). *P* values in panel **(A-E)** were determined by Kruskal-Wallis test. *P* values in panel **(F-H)** were determined by one-way ANOVA. *P < 0.05, **P < 0.01, ***P < 0.001 compared to the WT+LPS group.

To investigate in greater detail how *Stat3* influences the function of Th17 cells in periodontitis, primary CD4^+^T cells from cKO mice and WT mice were isolated and treated with *P. gingivalis* LPS. Compared with CD4^+^T cells from cKO mice, those from WT mice exhibited increased the mRNA expression of *Il17, Il6, Il21*, *Stat3* and *Rorγt* following treatment with *P. gingivalis* LPS, indicating that deletion of *Stat3* disrupted the expression of Th17-related cytokines and transcriptional factors (**Figures 6A-E**). Moreover, the results of flow cytometric analysis demonstrated that the Th17/Treg ratio was lower in CD4^+^T cells from cKO mice than in CD4^+^T cells from WT controls in response to *P. gingivalis* LPS exposure (**Figures 6F-H**), which was consistent with the Th17 cell dysfunction observed *in vivo*.

### 3.7 Effects of *Stat3*-mediated Th17 cells on co-cultured BV2 cells

**Figure 7:**
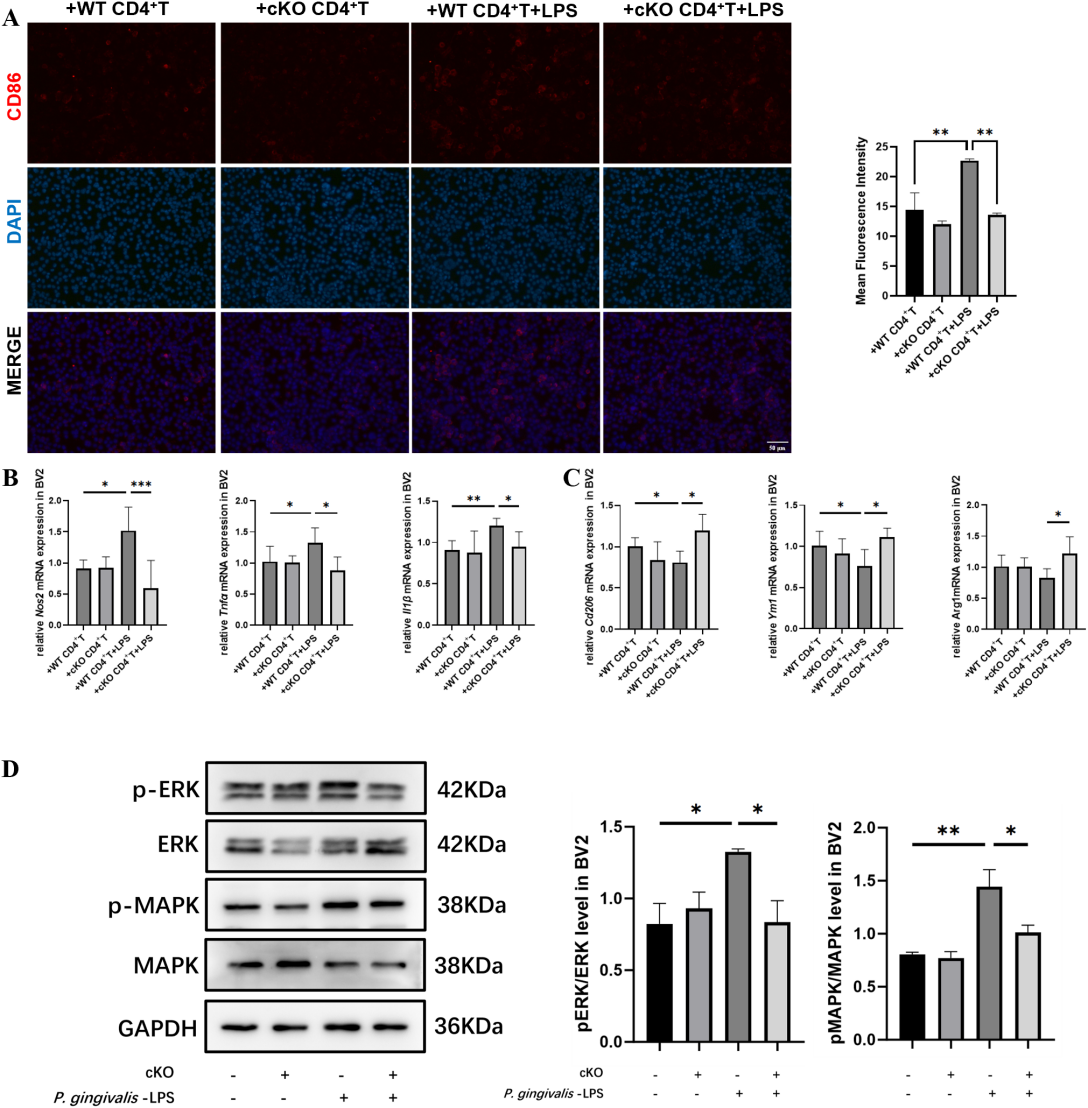
Effects of *Stat3*-mediated Th17 cells on co-cultured BV2 cells. **(A)** Representative immunofluorescence images of CD86 (red) staining in BV2 cells and mean fluorescence intensity of CD86-positive cells (Scale bar=50 μm) (*n*=5 per group). **(B)** mRNA expression of M1 polarization markers (*Nos2, Tnfα* and *Il1β*) in BV2 cells after treated with CM for 24 h (*n*=4 per group). **(C)** mRNA expression of M2 polarization markers (*Cd206, Ym1* and *Arg1*) in BV2 cells after treated with CM for 24 h (*n*=4 per group). **(D)** Representative western blot bands and quantitative analysis of ERK, p-ERK, MAPK and p-MAPK in BV2 cells after treated with CM for 24 h (*n*=3 per group). *P*-values were determined by one-way ANOVA. *P < 0.05, **P < 0.01, ***P < 0.001 compared to the +WT CD4^+^T +LPS group. CM, conditional medium.

Finally, we established an *in vitro* co-culture system by culturing BV2 cells with CM obtained from CD4^+^T cells to verify our hypothesis that Stat3 was involved in the interaction between Th17 and microglia. CD86-positive cells significantly increased in the +WT CD4^+^T+LPS group compared with that in the +WT CD4^+^T group (**Figure 7A**). Similarly, the mRNA expression of M1-related genes, including *Nos2, IL1β* and *Tnfα* increased, whereas those of M2-related genes decreased in the +WT CD4^+^T+LPS group (**Figures 7B, C**). These pro-inflammatory responses were mitigated in the +cKO CD4^+^T+LPS group. Western blot analysis revealed that p-ERK and p-MAPK protein levels elevated in the +WT CD4^+^T+LPS group compared with that in the +WT CD4^+^T group. The activation of ERK/MAPK signaling pathway was inhibited in the +cKO CD4^+^T+LPS group (**Figure 7D**) and in presence of an ERK inhibitor (**Supplementary Figure S3**).

## 4 Discussion

In this study, we found that patients with cognitive impairment presented worse periodontal conditions and higher levels of Th17-related cytokines in GCF than cognitively normal participants. We further established a periodontitis model through injection of *P. gingivalis* LPS in mice with conditional deletion of *Stat3* in their Th17 cells (Data of gene knockout validation not shown). Obvious impairment in learning and memory abilities associated with Th17/Treg imbalance and neuroinflammation was observed in the WT-LPS group. However, in the cKO-LPS group, these manifestations were significantly mitigated, as was the Th17 pathogenicity resulting from *P. gingivalis* LPS-induced periodontitis. This indicated that *Stat3* was involved in how Th17 cells influence the correlation between periodontitis and cognitive impairment.

Clinical studies have shown that patients with cognitive impairment tend to exhibit poorer oral hygiene and more severe periodontal damage (24, 25). In this study, we found that the PD and CAL were higher in the CI group than in the CN group. A cross-sectional study revealed increased serum levels of TNF-α and IL-6 in patients with cognitive impairment and periodontitis (26), which suggested that the presence of systemic inflammation in periodontitis patients may affect the progression of neurodegenerative diseases. Analysis of GCF is considered an important tool for the detection of molecular biomarkers associated with periodontitis (27). Given that the GCF composition may to some extent reflect systemic circulation, it may provide insight into the relationship between periodontitis and systemic conditions (28). Here, we tested the hypothesis that cognitive impairment was associated with an increased periodontal inflammatory response mediated by Th17 cells. This possibility was supported by the increase in the levels of Th17-related cytokines in GCF from the CI group. Combined with our previous study (16), we suspected that the inflammatory responses mediated by Th17 cells played a key role in the mouth-to-brain axis.

It has been demonstrated that Th17-mediated responses are associated with the progression of periodontitis. Levels of Th17-related cytokines, such as IL-17, IL-23 and IL-21 were found upregulated in gingival tissues of periodontitis patients, and the levels of these proinflammatory cytokines were shown to be correlated with the severity of alveolar bone destruction (29, 30). Similarly, in experimental periodontitis models, Th17 cells have also been found to be activated and drive inflammation and bone destruction (31). Periodontal pathogenic bacteria and their virulence factors can trigger the proliferation and cytokine secretion by Th17 cells, which in turn, promote the production of osteoclastic mediators and induce gingival epithelial cells to produce pro-inflammatory cytokines (32, 33). In animal models, inhibition of STAT3 or RORγt in CD4^+^T cells resulted in the suppression of gingivitis and alveolar bone resorption (34, 35). Consistent with these findings, our findings demonstrated that alveolar bone destruction and gingivitis induced by injection of *P. gingivalis* LPS were alleviated in mice with conditional knockout of *Stat3* in Th17 cells.

While it is increasingly clear that an association exists between AD and periodontitis, the causal mechanism underlying the correlation remains unclear. It is presumed that periodontitis leads to inflammation not only locally within the oral cavity but also systemically. In addition, increasing evidence suggests that periodontitis also aggravates neuroinflammation, thereby contributing to the pathogenesis of neurodegenerative diseases (36, 37). It is widely accepted that neuroinflammation is mediated by microglia and astrocytes, while CD4^+^T cells have been reported to play a significant role in regulating neuroinflammation (38, 39). Both *in vivo* and *in vitro* studies have demonstrated that Th17 cells and their signature cytokine IL-17 can disrupt the function of the blood-brain barrier (BBB), which plays a key role in isolating the CNS from the systemic circulation to sustain an optimal microenvironment (40–42). In our study, an increased infiltration of Th17 cells was observed both in the periphery and the CNS in the WT-LPS group, thereby initiating or exacerbating neuroinflammatory responses. However, numerous studies have confirmed that *P. gingivalis* LPS and pro-inflammatory cytokines could compromise the integrity of the BBB, which allows the peripheral pro-inflammatory mediators to enter the brain (40, 43), and our research cannot rule out the influence of these peripheral proinflammatory mediators on neuroinflammation. Therefore, we believe that the Th17 pathogenicity induced by periodontitis can affect microglia activation in two ways. On one hand, it indirectly activated microglia by upregulating proinflammatory cytokines in the peripheral, which then crossed the BBB and transmit inflammatory signals to microglia. On the other hand, Th17 cells themselves could infiltrate into the CNS, activating microglia by secreting characteristic proinflammatory cytokines such as IL-17A.

A shift in the Th17/Treg balance toward Th17 cells is a significant contributor to the development of inflammatory diseases, and this shift is primarily regulated by *Stat3*. *Stat3* plays a key role in initiating Th17 cell differentiation, cell proliferation and IL-17A expression through upregulation of RORγt expression (44, 45). IL-6 is known to activate *Stat3* signaling pathway, which, in turn, promotes the production of proinflammatory cytokines, including IL-17 and IL-21 (46). In our study, primary CD4^+^T cells were isolated to investigate the effects of *Stat3* knockout on Th17 cells *in vitro*. We found that the expression of IL-17, IL-21 and IL-6 decreased in the cKO+LPS group, indicating the dysfunction in Th17 cells in response to *P. gingivalis* LPS. Specifically, IL-21, expressed by Th17 cells, has been identified as a T cell growth factor important for T-cell activation and proliferation (47). Furthermore, both Th17 cells and IL-17A can promote microglial activation by binding to their receptors, thereby enhancing oxidative stress and the release of proinflammatory cytokines (48, 49). Our results showed that in absence of *Stat3* in Th17 cells, the mRNA expression of *Rorγt* was not upregulated, which suppressed Th17 differentiation. Consequently, Treg differentiation, which was inhibited in the WT+LPS group, was enhanced in the cKO+LPS group, leading to the restoration of Th17/Treg balance, thereby protecting the CNS from an exaggerated inflammatory response.

Additionally, microglial polarization toward M1 phenotype was observed when co-cultured with CM from CD4+ T cells in our study. The pro-inflammatory pathways related to microglia activation are downstream targets of the MAPK/ERK signaling pathway, indicating that MAPK/ERK is critical to generating pathological neuroinflammation (50). It has been reported that LPS can induce a pro-inflammatory response in microglia by activating the MAPK/ERK signaling pathway, however, whether the interaction between CD4^+^T cells and microglia influences this pathway remains unclear (51). To test this possibility, MAPK/ERK phosphorylation levels were examined in BV2 cells following co-culture with CM from CD4^+^T cells. The results showed that phosphorylation levels of MAPK and ERK increased in BV2 cells cocultured with the +WT CD4^+^T+LPS group, whereas the activation of the pathway was inhibited in BV2 cells treated with an ERK inhibitor or co-cultured with the +cKO CD4^+^T+LPS group. Studies have reported that microglia express IL-17A receptors, implying that Th17 cells may induce microglial activation (52, 53). These results suggested that the activation of the adaptive immune response in the CNS related to Th17 cells might be attributed to inflammatory mediators from the mouth-to-brain axis.

Our study had some limitations. First, the clinical findings in this study could only suggest a potential correlation between periodontitis and cognitive impairment, which could not confirm causality. Cohort studies with larger sample sizes and longer follow-up periods are needed to establish a causal association. Second, a morphological evaluation based on fractal and skeleton analyses may be preferred, which may provide an improved qualitative assessment of microglial activation.

In conclusion, our data provide evidence supporting that Th17 cells may play a role in the connection between periodontitis and cognitive impairment by sustaining systemic inflammation and inducing microglial activation. The conditional deleting of *Stat3* in Th17 cells alleviated cognitive impairment in periodontitis mice, likely through the mitigation of Th17 pathogenicity and microglial inflammatory responses. These results suggest that *Stat3*-mediated Th17 pathogenicity could serve as a novel perspective for exploring the mechanisms underlying the mouth-brain axis.

## Data Availability

The datasets presented in this study can be found in online repositories. The names of the repository/repositories and accession number(s) can be found in the article/**Supplementary Material**.
