# Supplementary material for: Stat3-mediated Th17 pathogenicity induced by periodontitis contributes to cognitive impairment by promoting microglial M1 polarization
Source: Front Immunol. 2025 Aug 26;16:1590665. doi: 10.3389/fimmu.2025.1590665 (PMC12417192; doi:10.3389/fimmu.2025.1590665)
Supplement: Supplementary file 1 [file DataSheet1.docx]

Supplementary Material

# Supplementary Data

https://www.jianguoyun.com/p/DQGZ0agQ_7CnDRix3u8FIAA

# Supplementary Figures and Tables

## Supplementary Tables

**Supplementary Table 1.** Nucleotide sequences of primers used in this study

|  | **Primersequence(5’-3’)** | |
| --- | --- | --- |
| Gene | Forward | Reverse |
| *βactin* | GTGACGTTGACATCCGTAAAGA | GCCGGACTCATCGTACTC |
| *Il1β* | AACCTGCTGGTGTGTGACGTTC | CAGCACGAGGCTTTTTTGTTGT |
| *Il10* | CATTAATATTTAACGATGTGGATGCG | GCCTACCATCTTTAAACTGCACAAT |
| *Il17a* | TCAGCGTGTCCAAACACTGAG | CGCCAAGGGAGTTAAAGACTT |
| *Il21* | GGACCCTTGTCTGTCTGGTAG | TGTGGAGCTGATAGAAGTTCAGG |
| *Il6* | ATCCAGTTGCCTTCTTGGGACTGA | TAAGCCTCCGACTTGTGAAGTGGT |
| *Tnfα* | GACAAGGCTGCCCCGACTACG | CTTGGGGCAGGGGCTCTTGAC |
| *Stat3* | CCCCCGTACCTGAAGACCAAG | TCCTCACATGGGGGAGGTAG |
| *Rorγt* | AGTGTAATGTGGCCTACTCCT | GCTGCTGTTGCAGTTGTTTCT |
| *Nos2* | GTTCTCAGCCCAACAATACAAGA | GTGGACGGGTCGATGTCAC |
| *Mertk* | CAGGGCCTTTACCAGGGAGA | TGTGTGCTGGATGTGATCTTC |
| *Ym1* | CAGGTCTGGCAATTCTTCTGAA | GTCTTGCTCATGTGTGTAAGTGA |
| *Arg1* | CTCCAAGCCAAAGTCCTTAGAG | AGGAGCTGTCATTAGGGACATC |
| *Cd206* | CTCTGTTCAGCTATTGGACGC | CGGAATTTCTGGGATTCAGCTTC |

**Supplementary Table 2.** Participants’ demographic features and clinical characteristics

|  | CN(n=17) | CI (n=17) | *P*-value | *P*-value（Ajusted） |
| --- | --- | --- | --- | --- |
| **Demographics** |  |  |  |  |
| Age（mean±SD）years | 71.76±4.49 | 76.00±8.49 | / |  |
| Male (%) | 7 (41.18) | 6 (35.29) | / |  |
| Female (%) | 10 (58.82) | 11 (64.71) |  |  |
| **Neurological indices** |  |  |  |  |
| MoCA (mean±SD) | 27.44±1.31 | 13.00±5.03 | *＜0.0001* | *＜0.001* |
| **Periodontal indices** |  |  |  |  |
| Number of teeth | 25.18±4.71 | 17.73±9.16 | *＜0.01* | *＜0.05* |
| PPD (mean ± SD) mm | 2.61±0.86 | 3.28±0.76 | *＜0.05* | *＜0.05* |
| CAL (mean ± SD) mm | 3.54±0.99 | 5.09±1.40 | *＜0.01* | *＜0.01* |
| CAL>3 mm% (mean±SD) | 47.13±24.77 | 78.60±22.27 | *＜0.01* | *＜0.01* |
| BOP % (mean±SD) | 61.44±26.02 | 83.19±22.81 | *＜0.05* | *＜0.05* |

*P* values were determined by independent *t* test or ‌Mann-Whitney U test based on whether the data followed a normal distribution. Adjustment for age and sex was performed by linear regression analysis.

## Supplementary Figures


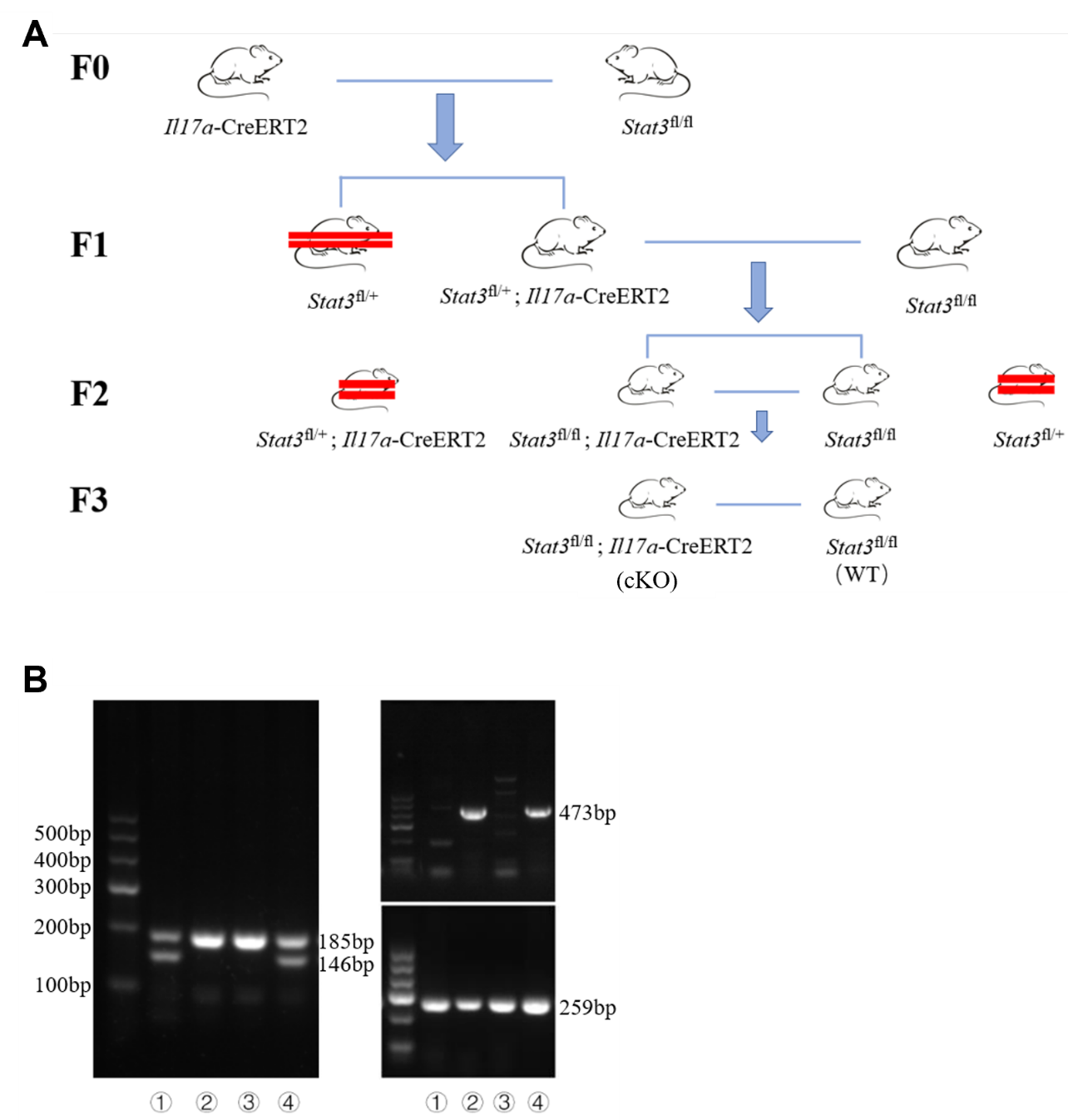


**Supplementary Figure 1.** Breeding and genotyping of conditional knockout mice (A) Breeding roadmap of *Stat3*^fl/fl^; *Il17a*-CreERT2 (cKO) mice and *Stat3*^fl/fl^ (WT) mice. (B) Genotyping of cKO mice by PCR and DNA agarose gel electrophoresis. ①*Stat3*^fl/+^；②*Stat3*^fl/fl^; *Il17a*-CreERT2 (cKO)； ③*Stat3*^fl/fl^ (WT); ④*Stat3*^fl/+^; *Il17a*-CreERT2.


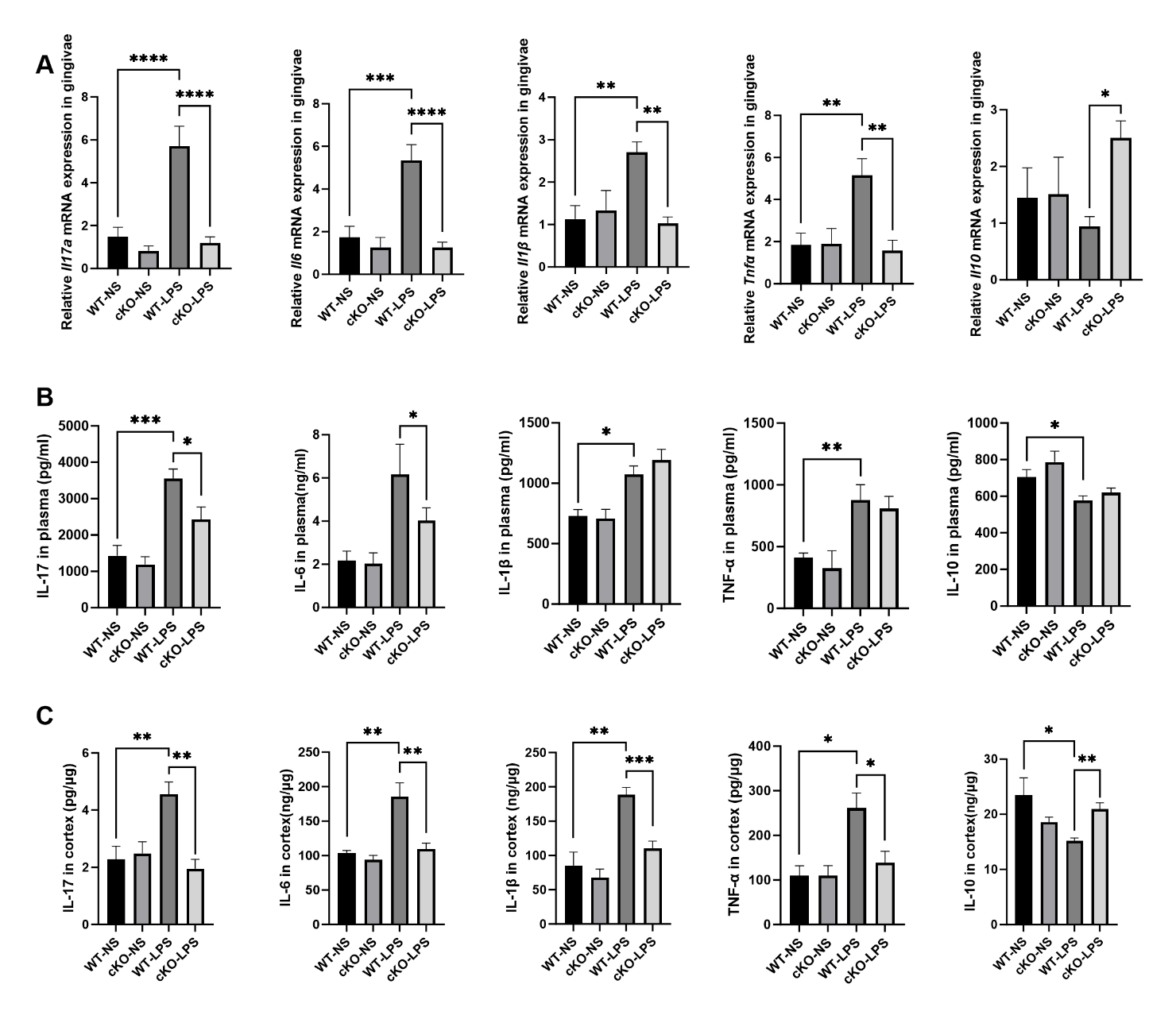


**Supplementary Figure 2.** **Effects of *Stat3*-mediated Th17cells on gingivitis, systemic inflammation and neuroinflammation in periodontitis mice.** The mRNA expression of pro-inflammatory cytokines (*Il1β, Il17a, Il6* and *Tnfα*) and anti-inflammatory cytokines (*Il10*) were detected by RT-qPCR in gingivae. The levels of these cytokines were measured by ELISA in plasma and cortex. (a) mRNA expression of inflammatory cytokines in gingivae (*n*=6 per group). (b) Protein levels of inflammatory cytokines in plasma (*n*=5 per group). (c) Protein level of inflammatory cytokines in cortex (*n*=5 per group). Data were presented as the mean ± standard error of mean. *P* values were determined by Kruskal-Wallis test. **P* <0.05, ***P* < 0.01, ****P* < 0.001, **** *P* < 0.0001 compared to the WT-LPS group


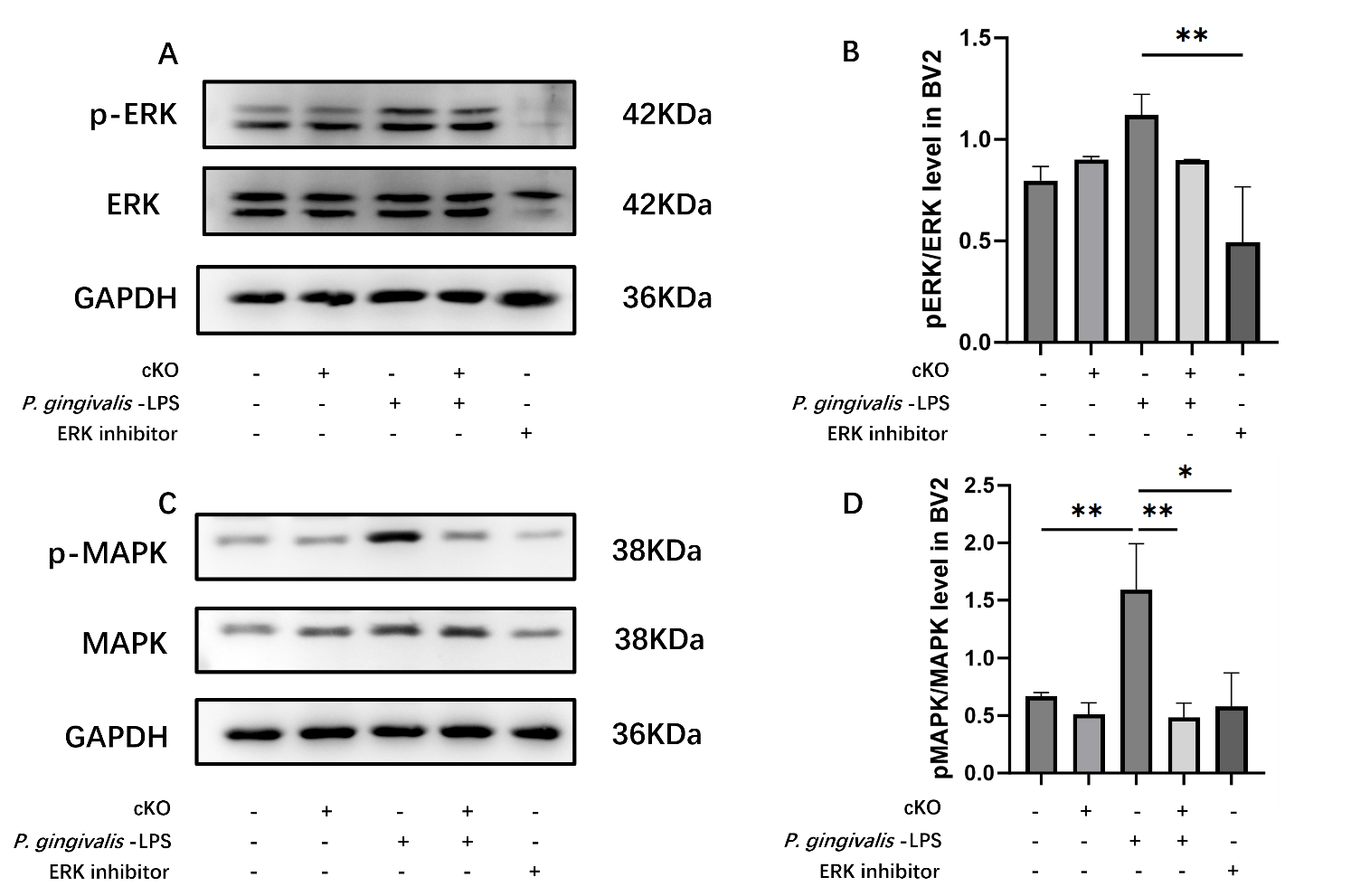


**Supplementary Figure 3. Effects of *Stat3*-mediated-Th17 cells on co-cultured BV2 cells.** **(A)** Representative western blot bands and **(B)** quantitative analysis of p-ERK/ERK in BV2 cells. **(C)** Representative western blot bands and **(D)** quantitative analysis of p-MAPK/MAPK in BV2 cells after treatment for 24 h (*n*=3 per group). *P*-values were determined by one-way ANOVA. **P* < 0.05, ***P* < 0.01, compared to the +WT CD4^+^T +LPS group.
